# Unraveling the Enigma of Aortic Dissection: From Genetics to Innovative Therapies

**DOI:** 10.7759/cureus.57803

**Published:** 2024-04-08

**Authors:** Swathi Godugu, Tanya Sinha, Mahendrarajah Pradeepan, Anthony Eze-odurukwe, Syed Faqeer Hussain Bokhari, Rabia Islam, Danyal Bakht, Hamza Islam, Muhammad Farhan

**Affiliations:** 1 General Medicine, Zaporizhzhia State Medical University, Zaporizhzhia, UKR; 2 Medical Education, Tribhuvan University, Kathmandu, NPL; 3 General Medicine, Lugansk State Medical University, Lugansk, UKR; 4 Surgery, Salford Royal NHS Foundation Trust, Manchester, GBR; 5 Surgery, King Edward Medical University, Lahore, PAK; 6 Medicine and Surgery, Faisalabad Medical University, Faisalabad, PAK; 7 Medicine and Surgery, Mayo Hospital, Lahore, PAK; 8 Internal Medicine, Punjab Medical College, Faisalabad, PAK; 9 Department of Medicine, Ajman University, College of Medicine, Ajman, ARE

**Keywords:** surgery, vascular, angiography, aortic rupture, review, management, aortic dissection

## Abstract

Aortic dissection (AD) presents a critical medical emergency characterized by a tear in the aortic wall, necessitating prompt recognition and management to mitigate catastrophic complications. Despite advancements in medical technology and therapeutic interventions, AD remains a formidable challenge, often resulting in severe morbidity and mortality. This narrative review provides a comprehensive overview of AD, encompassing its clinical presentation, diagnostic modalities, and management strategies, while also exploring emerging trends and innovations in its management. Genetic predispositions significantly influence AD pathogenesis, with over 30 contributory genes identified, emphasizing the importance of genetic screening and counseling. Classification systems such as Stanford and DeBakey, alongside their revised counterparts, aid in categorizing AD and guiding treatment decisions. Advancements in diagnostic imaging, including transesophageal echocardiography and computed tomography angiography, have enhanced diagnostic precision, augmented by artificial intelligence and machine learning algorithms. Pharmacological innovations focus on optimizing medical therapy, while surgical and endovascular approaches offer minimally invasive treatment options. Hybrid procedures and aortic valve-sparing techniques broaden treatment avenues, while bioresorbable stent grafts hold promise for tissue regeneration. Collaborative efforts and ongoing research are essential to address remaining challenges and improve outcomes in managing AD. This review contributes to the understanding of AD's complexity and facilitates informed decision-making in clinical practice, underscoring the imperative for continued innovation and research in AD management.

## Introduction and background

Aortic dissection is a life-threatening medical emergency characterized by a tear in the inner layer of the aorta, the largest artery in the human body. This condition leads to the formation of a false passage (false lumen) within the aortic wall, which can propagate along the length of the vessel and weaken the vascular structure [1]. If left untreated, AD can cause catastrophic complications such as organ malperfusion, stroke, or even death. AD can occur anywhere along the aorta, but it most commonly affects the thoracic aorta, particularly the ascending aorta [2]. This medical condition requires prompt recognition, an accurate diagnosis, and immediate management to improve patient outcomes. Physicians accurately suspect the diagnosis in only a minority of cases, ranging from 15% to 43% of confirmed instances of AD. Without prompt treatment, mortality rates can approach 50% within the first 48 hours of symptom onset. Despite extensive literature on the topic, a considerable number of ADs are still missed in the emergency department. Factors contributing to a high miss rate in diagnosis of AD include perceived mildness of symptoms, clinical symptoms suggesting alternative diagnoses like acute coronary syndrome (ACS), and lack of expected results such as a pulse deficit or widened mediastinum on chest X-ray [1,3-5]. Various risk factors have been associated with the development of AD, including hypertension, genetic predisposition, connective tissue disorders, atherosclerosis, and trauma [6]. AD poses a significant burden on public health due to its high mortality and morbidity rates. Despite advancements in medical technology and therapeutic interventions, AD remains a challenging condition to manage, often resulting in severe complications and long-term disability.

The purpose of this narrative review is to provide a comprehensive overview of AD, including its clinical presentation, diagnostic modalities, and management strategies. By synthesizing the available literature and current evidence, this review aims to enhance our understanding of AD and its impact on public health. Furthermore, this review will explore emerging trends in the management of AD and highlight areas for future research and intervention. Overall, this narrative review seeks to contribute to the body of knowledge surrounding AD and facilitate informed decision-making in clinical practice.

## Review

Genetics and AD

Genetics constitute a pivotal element in the pathogenesis of thoracic aortic aneurysm and dissection (TAAD), a potentially life-threatening condition characterized by the debilitation of the aortic wall, predisposing it to dilation, aneurysm formation, and the ominous prospects of dissection or rupture [7,8]. Inherited connective tissue disorders substantially augment the risk of AD. Among these conditions, Marfan syndrome, vascular Ehlers-Danlos syndrome, familial thoracic aortic aneurysms or dissections, and bicuspid aortic valve exemplify hereditary afflictions distinguished by structural aberrations in the aortic wall, thereby heightening the susceptibility to both aneurysm formation and dissection [9]. Nevertheless, it is now discernible that several other genetic anomalies can predispose individuals to TAAD, irrespective of the presence of conventional risk factors.

Presently, over 30 contributory genes have been elucidated, encompassing a diverse array of biological pathways implicated in the preservation of vascular integrity and functionality. For instance, mutations affecting genes encoding constituents of the extracellular matrix, such as FBN1, FBN2, COL3A1, and ELN, have the propensity to disrupt the structural robustness of the aortic wall, consequently fostering weakening and dilation. Correspondingly, mutations impacting vascular smooth muscle cell contractility, including MYH11, ACTA2, and MYLK, can impede the contractile efficacy of smooth muscle cells, thereby engendering dysfunction in the aortic wall and predisposing individuals to dissection [8,10,11]. Moreover, genetic aberrations concerning the transforming growth factor-ß (TGF-ß) signaling pathway, such as mutations in TGFBR1, TGFBR2, and SMAD genes, have been implicated in the pathogenesis of AD. Perturbations in TGF-ß signaling have the potential to disrupt the delicate equilibrium of extracellular matrix homeostasis, thereby engendering structural anomalies in the aortic wall and augmenting susceptibility to AD [10,11].

The dichotomous classification of TAAD into syndromic and non-syndromic forms underscores the heterogeneity of genetic influences on this malady. Syndromic forms, constituting a minority of TAAD cases, are typified by the involvement of extra-aortic organ systems and are often affiliated with well-defined genetic syndromes, such as Marfan syndrome and Loeys-Dietz syndrome. In contradistinction, non-syndromic forms encompass the preponderance of TAAD cases and are primarily confined to the aorta, devoid of overt involvement of other organ systems [7,8].

Genetic screening has emerged as a salient tool for identifying individuals at risk of AD and steering clinical management strategies. By discerning specific genetic mutations correlated with TAAD, genetic screening aids in stratifying patients based on their vulnerability to developing AD. Furthermore, genetic testing facilitates the premature detection of TAAD in asymptomatic individuals with a familial history of the condition, thereby enabling timely intervention and prophylactic measures [8]. The American College of Cardiology/American Heart Association proffers recommendations for genetic counseling and testing for first-degree relatives of patients harboring known genetic mutations associated with TAAD, such as FBN1, TGFBR1, TGFBR2, COL3A1, ACTA2, and MYH11 [8,12]. Additionally, sequencing of other genes may be contemplated in patients with a familial history of TAAD and clinical manifestations evocative of specific genetic mutations.

Classification systems

**Figure 1:**
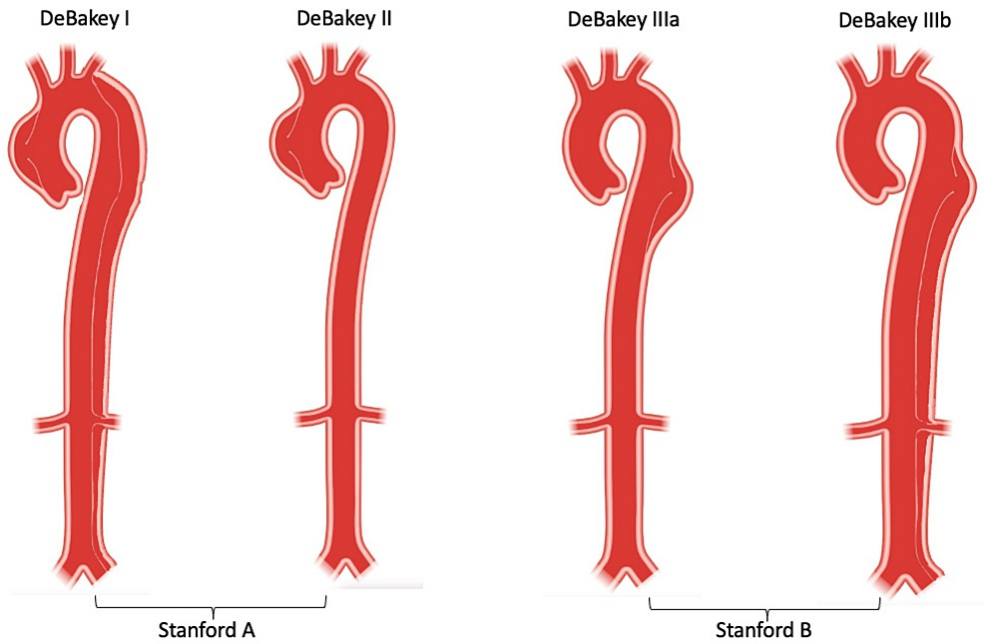
Stanford and DeBakey classification of aortic dissection. Adapted from Yuan et al. [13]

The traditional classification systems for AD include Stanford and DeBakey classification systems. The Stanford classification system is one of the most widely used systems for categorizing AD based on its anatomical involvement and clinical implications. This classification divides AD into two main categories, type A and type B. Type A dissection involves the ascending aorta, aortic arch, or both, potentially extending to the descending aorta. Type B dissection is confined to the descending thoracic aorta distal to the origin of the left subclavian artery. Type B dissection may extend into the abdominal aorta but does not involve the ascending aorta [1,13]. The DeBakey classification system provides a more comprehensive classification of AD based on the extent of the dissection and the involvement of aortic arch branches. This classification includes three categories, type I, II, and II. Type I dissection involves the ascending aorta, aortic arch, and descending aorta, extending distally beyond the origin of the left subclavian artery. Type II dissection is confined to the ascending aorta, without the involvement of the aortic arch or descending aorta. Type II dissection may extend proximally towards the aortic root or distally towards the aortic arch branches but does not involve the descending thoracic aorta. Type III dissection is confined to the descending thoracic aorta, distal to the origin of the left subclavian artery. Type III dissection may extend into the abdominal aorta but does not involve the ascending aorta or aortic arch (Figure 1) [1,14].

In addition to the Stanford and DeBakey classifications, several revised classification systems have been proposed to further refine the classification of AD and incorporate additional prognostic factors [15]. These revised classification systems may include additional categories such as atypical dissection, complicated dissection, or intramural hematoma, based on imaging findings, clinical presentation, and associated complications (Table 1). They include the International Registry of Aortic Dissection (IRAD) classification, Type-Entry-Malperfusion (TEM) classification, and SVS/STS 2020 reporting standards [16-18]. The revised classification systems enhance personalized management approaches for AD by categorizing patients according to the extent, location, and severity of their condition. This facilitates predicting outcomes, guiding treatment decisions, and optimizing patient care. Furthermore, these systems enable risk stratification in research and promote standardized reporting across various healthcare settings.

**Table 1:** Summary of aortic dissection classification systems. Adapted from Hamilton [15]

Stanford Classification	
Type A	Dissection of the ascending and descending aorta
Type B	Dissection of the descending aorta
De Bakey Classification	
Type 1	Dissection of the entire aorta
Type 2	Dissection of the ascending aorta
Type 3	Dissection of the descending aorta
New Classification	
Class 1	Classical aortic dissection with an intimal flap between true and false lumen
Class 2	Medial disruption with formation of intramural haematoma/haemorrhage
Class 3	Discrete/subtle dissection without haematoma, eccentric bulge at tear site
Class 4	Plaque rupture leading to aortic ulceration, penetrating aortic atherosclerotic ulcer with surrounding haematoma, usually subadventitial
Class 5	Iatrogenic and traumatic dissection
Class 1–5 Represent a subdivision to the Stanford or De Bakey classification	

Evolving phenotypes of AD

AD manifests across a continuum of symptomatology, spanning from classical to atypical presentations, thereby posing formidable challenges for prompt diagnosis. The divergent clinical presentation engenders diagnostic intricacies, potentially culminating in delays or erroneous diagnoses. Differential diagnoses encompass acute coronary syndrome, pulmonary embolism, aortic aneurysm rupture, esophageal perforation, acute pancreatitis, and musculoskeletal disorders [1].

The classical clinical depiction of AD is distinguished by an acute onset and intense, lacerating pain in the thorax or dorsum [19]. In contrast, atypical manifestations encompass abdominal discomfort, dyspnea, cough, dorsal pain, stroke-like symptoms, febrile episodes, and diaphoresis [20,21]. Patients afflicted with Type A dissection typically endure an abrupt commencement of thoracic pain typified by attributes of sharp or piercing quality, whereas those with Type B dissection often present with localized dorsal discomfort or discomfort radiating to the abdominal region, suggestive of extension to the distal segment of the aorta [13]. Nevertheless, a subset of dissection patients, comprising up to 6.4% of cases, may evince a paucity of pain, with predominant left-sided neurological deficits constituting the prevalent presenting symptom among them [13,22]. Pulse deficits, reported in up to 30% of patients, serve as highly indicative of AD, thereby warranting further investigative measures [13,22]. Hypotension and syncope assume particular gravity, signifying adverse prognostic implications and mandating expeditious intervention owing to underlying conditions such as cardiac tamponade and aortic rupture [13].

The misclassification of AD and ensuing delays in management can exert profound deleterious effects on patient outcomes due to the intrinsic nature of the pathology. Several diagnostic hurdles contribute to this phenomenon, encompassing the low prevalence of the condition, precipitously evolving pathology, enigmatic symptomatology, nonspecific clinical manifestations, potential conflation with alternative acute conditions such as acute coronary syndromes, and a dearth of specialized therapeutic infrastructure. Of particular concern is the similarity between AD and acute coronary syndromes, which heightens the risk of inappropriate therapeutic interventions. Moreover, the concurrence of AD with malperfusion syndromes both precipitates misdiagnosis and serves as an index of disease complications, thereby necessitating bespoke therapeutic stratagems in the emergency care setting [23]. Despite advancements in diagnostic modalities such as imaging techniques and biomarker utilization, misdiagnosis of AD remains endemic, with extant guidelines proffering limited prophylactic measures.

Advancements in diagnostics

Advancements in diagnostic methodologies for AD have engendered a paradigm shift in patient care, with a concerted emphasis on expedited and precise identification to catalyze timely therapeutic interventions. Historically, the clinical assessment of putative cases has been predicated on a confluence of historical inquiry, physical examination, and risk stratification, with manifestations such as acute-onset severe thoracic discomfort serving as sentinel indicators. Transthoracic echocardiography (TTE) has traditionally constituted a cornerstone as the initial imaging modality for evaluating patients with suspected AD due to its wide availability and bedside applicability. Despite its indispensable role, TTE encounters inherent limitations in adequately visualizing the ascending aorta and occasionally lacks the capability to conclusively ascertain the presence of dissection. Nevertheless, TTE excels in discerning associated complications such as aortic regurgitation, aortic dilation, the discernment of an intimal flap, or the identification of pericardial effusion, thus augmenting diagnostic precision in clinical practice [24]. Transesophageal echocardiography (TEE) stands out for its unparalleled ability to visualize the proximal aorta, encompassing the aortic root and ascending aorta, thereby surpassing the capabilities of TTE in terms of sensitivity and specificity for diagnosing AD. Notably, TEE excels in accurately delineating critical features such as the intimal flap and false lumen, crucial for precise diagnosis. Additionally, TEE exhibits remarkable efficacy in identifying associated complications like aortic regurgitation, thus rendering it indispensable in guiding emergent management strategies [25].

Whilst echocardiography retains its indispensable role, computed tomography angiography (CTA) has emerged as the paragon in the diagnostic armamentarium for AD, courtesy of its exalted sensitivity and specificity. CTA, by virtue of its expeditious acquisition and meticulous anatomical elucidation, serves as a vanguard in guiding both surgical and endovascular interventions [26]. Magnetic resonance angiography (MRA) and magnetic resonance imaging (MRI) proffer viable alternatives to CTA, particularly advantageous in cohorts evincing contraindications to iodinated contrast or renal insufficiency. The commendable soft tissue contrast of MRA and the comprehensive evaluative prowess of MRI conduce to the stratification of risk and the formulation of therapeutic blueprints [27].

Concomitantly, the amalgamation of artificial intelligence (AI) and machine learning (ML) algorithms has significantly advanced diagnostic accuracy. ML algorithms, trained on datasets of comparable quality, possess the capability to automatically extract imaging features with a precision equivalent to manual annotations, thereby enhancing diagnostic effectiveness. Within the realm of ML, deep learning (DL) has demonstrated exceptional proficiency in tasks such as image classification and segmentation. In the context of AD imaging, DL algorithms showcase remarkable aptitude in recognizing patterns within clinical and imaging datasets, thereby augmenting human observation and contributing to improved diagnosis and risk assessment [28]. Cutting-edge methodologies, exemplified by the integration of deep learning with automated morphological analysis, have heralded a new era of diagnostic accuracy, resulting in enhancements in diagnostic reliability, even in scenarios where conventional approaches may encounter challenges, such as the absence of contrast-enhanced CT scans [28,29].

Concurrently, biomarkers have emerged as promising complements to traditional imaging modalities, providing insightful perspectives into disease diagnosis, prognosis, and pathogenesis. Proteins, RNA, and DNA markers offer a multifaceted interpretation of AD. Protein markers like D-dimer aid in excluding AD within the initial 24-hour period, while inflammatory markers such as NLRP3 and interleukins contribute to the rapid detection of the condition. Cardiac markers such as N-terminal pro b-type natriuretic peptide (NT-proBNP) act as indicators of diagnosis, reflecting the degree of myocardial involvement. RNA markers, including microRNAs and circMARK3, exemplify the potential of molecular indicators in diagnosis, thereby offering nuanced insights into disease pathogenesis. Additionally, cell-free DNA (cfDNA) derived from plasma provides a minimally invasive complement to standard diagnostic modalities, thereby enriching our understanding of AD [30].

Notwithstanding these challenges, the symbiotic amalgamation of traditional imaging modalities, AI, ML, DL, and biomarkers portends an epoch of heightened diagnostic precision, efficacy, and personalized therapeutic paradigms for AD, ultimately accruing to the amelioration of patient outcomes and survival metrics.

Innovative therapeutic approaches

The management of AD remains a complex challenge despite significant progress. Fortunately, continuous advancements are revolutionizing both medical and interventional approaches, offering hope for improved outcomes. This section explores key areas of innovation related to the pharmacological, surgical and endovascular management of AD.

Pharmacological Innovations

Pharmacological innovations have played a significant role in the management of AD, particularly in optimizing medical therapy and preventing disease progression. Novel antihypertensive agents such as angiotensin receptor-neprilysin inhibitors and mineralocorticoid receptor antagonists have shown promise in reducing blood pressure and aortic wall stress in patients with AD. These agents may offer additional benefits beyond traditional beta-blockers or angiotensin-converting enzyme (ACE) inhibitors in certain patient populations such as additional protection against aortic wall remodeling and progression of dissection [31,32]. Vasodilators such as nitric oxide donors or phosphodiesterase inhibitors may help reduce aortic wall tension and improve myocardial oxygenation in patients with acute AD. These agents may be used as adjunctive therapy to beta-blockers or calcium channel blockers in select cases [33]. Emerging evidence suggests that inflammation plays a significant role in the pathogenesis of AD, leading to endothelial dysfunction, matrix degradation, and vascular remodeling [34]. Anti-inflammatory agents such as statins, corticosteroids, or monoclonal antibodies targeting pro-inflammatory cytokines may attenuate inflammation and prevent disease progression in patients with AD. While still in its early stages, gene therapy holds promise for correcting genetic abnormalities underlying connective tissue disorders that predispose individuals to dissection. This could prevent future events and improve overall patient management.

Surgical Innovations

Advances in surgical techniques and approaches have expanded the treatment options available for patients with AD, particularly in terms of improving surgical outcomes and reducing perioperative complications. Minimally invasive techniques such as endovascular stent grafting or robotic-assisted surgery offer potential advantages over traditional open surgical repair, including reduced surgical trauma, reduced incision size, shorter hospital stays, and faster recovery times. These techniques may be particularly beneficial in select patients with uncomplicated Type B dissection or high surgical risk. Hybrid approaches combining open surgical techniques with endovascular interventions have emerged as a promising treatment strategy for complex ADs involving the arch vessels or aortic root [35]. Hybrid procedures may allow for customized treatment options tailored to individual patient anatomy and pathology, optimizing outcomes while minimizing procedural risks. Aortic valve-sparing procedures such as the David procedure or the Yacoub procedure offer an alternative to traditional aortic valve replacement in patients with aortic root involvement [36]. These techniques preserve the native aortic valve while addressing concomitant aortic pathology, reducing the need for lifelong anticoagulation, and preserving ventricular function.

Endovascular Innovations

Endovascular innovations have revolutionized the management of AD, offering less invasive treatment options and expanding the eligibility criteria for endovascular repair. Newer stent grafts with improved flexibility and conformability are being developed to better adapt to complex aortic anatomies, offering more precise repair and potentially reducing complications. Fenestrated and branched stent grafts allow for precise positioning and sealing of complex ADs involving the aortic arch or visceral branches [37]. Bioresorbable stent grafts composed of biocompatible materials offer the potential for tissue regeneration and remodeling in patients with AD. These devices gradually degrade over time, allowing for physiological adaptation of the aorta and reducing the risk of long-term complications such as stent graft migration or infection [38,39]. Advancements in endovascular techniques allow for improved and minimally invasive management of branch vessel involvement in dissections, ensuring adequate blood flow to vital organs. Image-guided navigation systems using advanced imaging modalities such as fluoroscopy, intravascular ultrasound (IVUS), or optical coherence tomography (OCT) allow for precise placement and deployment of endovascular devices in patients with AD [40]. Real-time imaging guidance enhances procedural accuracy and reduces the risk of procedural complications, improving patient outcomes.

Challenges and future directions

Several challenges and opportunities for future research remain in improving clinical outcomes and reducing the substantial morbidity and mortality associated with AD. Future research should focus on uncovering additional genetic variants and elucidating their functional implications, paving the way for more comprehensive genetic screening and counseling. Moreover, integrating genomic data with other patient-specific factors, such as clinical characteristics and imaging findings, could enable the development of personalized risk prediction models and tailored treatment strategies. Although several promising biomarkers have emerged, their clinical utility remains limited due to insufficient validation and standardization. Large-scale, multicenter studies are needed to establish robust biomarker panels that can reliably aid in early diagnosis, prognostication, and monitoring of AD. Additionally, exploring novel biomarker classes, such as extracellular vesicles and metabolomics profiles, may yield insights into disease mechanisms and potential therapeutic targets. While imaging modalities like CTA and MRA have revolutionized AD diagnosis, challenges persist in accurately assessing aortic wall integrity, predicting disease progression, and identifying patients at high risk of complications. Future efforts should focus on developing advanced imaging techniques, such as molecular imaging and functional imaging, to provide a more comprehensive evaluation of aortic pathology. Additionally, leveraging AI and ML algorithms could enhance diagnostic accuracy, automate risk stratification, and optimize treatment planning.

Gene therapy holds promise for correcting genetic defects underlying connective tissue disorders, potentially preventing or reversing aortic pathology. Additionally, tissue engineering and regenerative medicine approaches, such as bio-engineered vascular grafts or cell-based therapies, could offer novel solutions for aortic repair and reconstruction. Furthermore, exploring the potential of targeted molecular therapies, such as inhibitors of dysregulated signaling pathways or modulators of inflammation, may yield new avenues for medical management of AD. Despite advances in acute management, AD often leads to long-term morbidity and diminished quality of life. Future research should prioritize patient-reported outcomes and investigate strategies to optimize functional recovery, psychological well-being, and reintegration into society. Additionally, developing comprehensive care models that integrate multidisciplinary teams, patient education, and supportive care could enhance overall patient experience and long-term outcomes.

## Conclusions

Aortic dissection stands as a critical medical emergency with potentially devastating consequences if not promptly identified and managed. Despite strides in medical technology and therapeutic interventions, AD remains a formidable clinical challenge, often resulting in severe complications and long-term disability. Classification systems such as the traditional Stanford and DeBakey classifications, alongside their revised counterparts, provide valuable frameworks for categorizing AD, guiding treatment decisions, and predicting outcomes. Genetic factors play a pivotal role in AD's pathogenesis, with over 30 contributing genes identified thus far. The presence of syndromic and non-syndromic forms underscores the diversity of genetic influences on this condition, emphasizing the significance of genetic screening and counseling in clinical practice. Furthermore, advancements in diagnostic imaging techniques, including transesophageal echocardiography and computed tomography angiography, have significantly enhanced our ability to accurately diagnose AD. The integration of artificial intelligence and machine learning algorithms, coupled with biomarker utilization, holds promise for further enhancing diagnostic precision and tailoring therapeutic approaches. Pharmacological, surgical, and endovascular innovations present new avenues for optimizing medical therapy, minimizing perioperative complications, and broadening treatment options for AD patients. Innovative therapeutic strategies, such as gene therapy, minimally invasive surgical techniques, and bioresorbable stent grafts, represent significant strides toward improving patient outcomes and survival rates. Nonetheless, ongoing research efforts and collaborative endeavors are indispensable to address the remaining challenges in effectively managing AD.
